# Ten Points to Improve Reproducibility and Translation of Animal Research

**DOI:** 10.3389/fnbeh.2022.869511

**Published:** 2022-04-21

**Authors:** Rainer Spanagel

**Affiliations:** Institute of Psychopharmacology, Central Institute of Mental Health, Medical Faculty Mannheim, Heidelberg University, Mannheim, Germany

**Keywords:** open science, p-hacking, HARCKing, confirmatory animal study, exploratory animal study, DSM-based animal model, 3R, 6R

## Abstract

Findings from animal experiments are often difficult to transfer to humans. In this perspective article I discuss two questions. First, why are the results of animal experiments often so difficult to transfer to humans? And second, what can be done to improve translation from animal experiments to humans? Translation failures are often the result of poor methodology. It is not merely the fact that low statistical power of basic and preclinical studies undermine a “real effect,” but the accuracy with which data from animal studies are collected and described, and the resulting robustness of the data is generally very low and often does not allow translation to a much more heterogeneous human condition. Equally important is the fact that the vast majority of publications in the biomedical field in the last few decades have reported positive findings and have thus generated a knowledge bias. Further contributions to reproducibility and translation failures are discussed in this paper, and 10 points of recommendation to improve reproducibility and translation are outlined. These recommendations are: (i) prior to planning an actual study, a systematic review or potential preclinical meta-analysis should be considered. (ii) An *a priori* power calculation should be carried out. (iii) The experimental study protocol should be pre-registered. (iv) The execution of the study should be in accordance with the most recent ARRIVE guidelines. (v) When planning the study, the generalizability of the data to be collected should also be considered (e.g., sex or age differences). (vi) “Method-hopping” should be avoided, meaning that it is not necessary to use the most advanced technology but rather to have the applied methodology under control. (vii) National or international networks should be considered to carry out multicenter preclinical studies or to obtain convergent evidence. (viii) Animal models that capture DSM-5 or ICD-11 criteria should be considered in the context of research on psychiatric disorders. (ix) Raw data of publication should be made publicly available and should be in accordance with the FAIR Guiding Principles for scientific data management. (x) Finally, negative findings should be published to counteract publication bias. The application of these 10 points of recommendation, especially for preclinical confirmatory studies but also to some degree for exploratory studies, will ultimately improve the reproducibility and translation of animal research.

## Introduction

Research in the field of behavioral neuroscience and science in general has a problem: many published scientific findings cannot be replicated. Most researchers are familiar with the situation in which a result from an experiment carried out years ago can no longer be reproduced. After a few sleepless nights and a lot of troubleshooting, however, a small methodological problem is typically identified and the results from former experiments can again be reproduced. Not only can the replication of one’s own results cause difficulties, but often other working groups cannot reproduce the results of their colleagues. Given this dilemma, it is for many researchers the greatest scientific satisfaction if another research group can replicate their published results. Sir Karl Popper, an Austrian-British philosopher who founded the philosophy of critical rationalism put this phenomenon in a nutshell: **“**We *don***’***t even take our own observations seriously or accept them as scientific observations until we have repeated and tested them. It is only through such repetitions that we can convince ourselves that it is not just an isolated* **“***coincidence***”** *that is involved, but rather events that are fundamentally inter-subjectively verifiable due to their regularity and reproducibility***”** (Popper, 1935). Popper**’**s critical rationalism is more relevant today than ever before. His philosophical approach represents a balanced middle ground between a belief in science and the relativism of truth. The replication of a scientific finding supports a belief in science, but if a finding is not replicated, it does not necessarily mean that the original finding is wrong, only that it needs to be relativized and subjected to critical discourse. This critical discourse usually leads to an explanation of the discrepancy, thereby sometimes placing opposing findings in relation and thus leading to better knowledge gain. The discourse, by way of critical rationalism, is not only the compass to maneuver through the corona crisis, but also a guide through the replication and translation crisis and runs like a red line through the following paragraphs of this perspective paper.

## The Replication Crisis is a Multidisciplinary Phenomenon and Contributes Largely to the Translation Failures from Animal to Human Research

How can one measure the replication of scientific findings? There is no generally accepted standard method for measuring and evaluating a replication. However, reproducibility is well-assessable using significance and *P*-values, effect sizes, subjective ratings, and meta-analyses of effect sizes. These indicators correlate with each other and together provide a meaningful statement about the comparison between the replication and the original finding (Open Science Collaboration, 2015). However, in animal studies, statistical analyses and resulting *P*-values underlie a wide sample-to-sample variability and therefore should not be solely used as the guiding principle to assess reproducibility (Halsey et al., 2015).

Replication failures are an inherent problem of doing science, but are we in a replication crisis? Indeed, meta-research that itself uses scientific methodology to study the quality of a scientific approach has identified widespread difficulty in replicating results in all experimental scientific fields, including behavioral neuroscience. This problem is termed “the replication crisis.” Although the term “crisis” has a subjective negative meaning, a recent survey of 1,500 researchers supports this general perception (Baker, 2016). Only the subjective assessment of replication was used as an indicator, and more than half of the respondents answered “Yes—there is a significant replication crisis” in research, and another almost 40% of respondents indicated that there is indeed a crisis. The term “replication crisis” is also often associated with the accusation of unethical behavior to data manipulation and the pure fabrication of results. The question of the extent of scientific misconduct inevitably arises. Fanelli (2009) conducted a systematic review and meta-analysis on this question. In anonymous surveys, scientists were explicitly asked whether they had ever fabricated or falsified research data or whether they had changed or modified results in order to improve the result. The meta-analysis of these studies showed that around 2% of those questioned admitted to scientific misconduct, the number of unreported cases is likely to be somewhat higher. It remains to be seen whether these 2% black sheeps show systemic misconduct resulting from the Publish or Perish incentive system, or whether this represents the normal range of unethical human behavior. If it is not scientific misconduct that leads to the replication crisis how does the problem of the lack of reproducibility of published results, which greatly affects the reliability of animal experimentation, come about?

## A Summary of Reasons that Contribute to Replication Failures in Animal Experiments

Many factors contribute to the replication failures of findings derived from animal research.

Most animal studies in the field of behavioral neuroscience are exploratory in nature and have a very low statistical power. A study with low statistical power has a reduced chance of detecting a true effect. Low power also reduces the likelihood that a statistically significant result reflects a true effect (Button et al., 2013). This results in a lack of repeatability with a wide sample-to-sample variability in the *P*-value. Thus, unless statistical power is very high (and much higher than in most experiments), the fickle *P*-value should be interpreted cautiously (Halsey et al., 2015). This fundamental problem of small sample sizes and the hereof resulting wide sample-to-sample variability in the fickle *P*-value largely contributes to replication failures.

Related to this inherent statistical challenge is the phenomenon of “p-hacking,” the selective reporting of significant data. P-hacking can be conducted in many ways. For example, by performing many statistical tests on the data and only reporting those that come back with significant results, by deciding to include or drop outliers, by combining or splitting treatment groups, and other ways to manipulate data analysis. Head et al. (2015) demonstrated how one can test for p-hacking when performing a meta-analysis and that, while p-hacking is probably common, its effect seems to be weak relative to the real effect sizes being measured.

HARKing, or Hypothesizing After the Results are Known, is also a source of uncertainty (Kerr, 1998). Although a study is usually planned using a working hypothesis, oftentimes the results neither support nor reject the working hypothesis, but may point to a new working hypothesis—the article is then written in a way that makes the most sense of the results.

Although p-hacking and HARKing are common practices in science, both likely have little impact on the replication crisis as compared to another problem: poor methodology. Thus, the accuracy with which animal research is collected and described, and the resulting robustness of the data, is generally very low (Perrin, 2014; Bespalov et al., 2016). Kilkenny et al. (2009) examined this systematically in 271 published animal studies. More than half of the studies did not provide information on the age of the animals, 24% of the studies did not provide information on the sex, 35% of the studies did not provide information on the number of animals used, 89% of the studies did not perform any randomization, and 83% of the studies did not provide information on blinding of the experiments (Kilkenny et al., 2009).

A problem that also relates to poor methodology that has yet to receive attention is a phenomenon it is referred to here as “method hopping.” Method hopping is the race for the newest technology. There is an extremely rapid development and turnover rate in technology especially in the neuroscience field. A technology that is currently considered state of the art can already be outdated a year later. This problem is escalated by the policies of top journals and many grant institutions, which always favor the use of the newest technology to answer a research question. An indication that method hopping contributes to replication failures is the fact that publications in high-profile journals such as Nature and Science that could not be replicated are cited 153 times more often than those studies that could be successfully replicated (Serra-Garcia and Gneezy, 2021). It is suggested that more-cited papers have more “interesting” results driven by new technologies which leads to a subjectively more lax peer-review process and subsequently to a negative correlation between replicability and citation count (Serra-Garcia and Gneezy, 2021). Hence, this race for the newest technology comes at a high price, as oftentimes researchers do not have their newest technology under control, and published measurements may subsequently rely on simple technical failures.

In addition to poor methodology, another overlooked problem is that published findings can ultimately only be generalized to a limited extent. The problem of generalization of findings from animal experiments is evident to all animal experimenters. In rodent studies, for example, strain, age, sex, and microbiome composition are critical biological factors that largely contribute to study differences. Numerous environmental factors such as light, temperature, cage size, numbers of animals per cage, enrichment, and food composition—to name just the most important ones—also contribute to large data variability. However, even if biological and environmental factors are standardized, findings can still differ widely. This is best exemplified by a multi-site study by John Crabbe from Portland together with colleagues from labs in Edmonton, Canada, and Albany, New York. They studied eight inbred strains of mice in all three labs using various behavioral tests (Crabbe et al., 1999). All laboratory conditions—from the light-dark cycle to the manufacturer of the mouse food, etc.—were harmonized between the three laboratories. However, the results were strikingly different across laboratories. For example, in a standard locomotor activity test using an identical open field apparatus, all strains in the different laboratories showed different activity measurements. This comparative study of locomotor activity in mice leads to the following conclusion: there are significant differences in locomotor activity between different inbred strains and these differences do not translate from one laboratory to another. The same conclusions can be drawn for alterations in body weight. All inbred strains differed significantly in weight gain; however, these differences could not be extrapolated from one laboratory to another. In summary, this suggests that results from one laboratory are not transferrable to another. Mice from an inbred strain that are per definition genetically identical exhibit different locomotor activity and weight gain in an Albany lab than in a Portland or Edmonton lab. However, sex differences were comparable across sites. In all 8 animal strains and in all three laboratories, female animals showed higher activity and lower weight gain than male animals. It is therefore possible to generalize that regardless of the inbred strain and laboratory in which locomotor activity is measured, female animals are generally more active and have a lower weight gain than male animals.

The study by Crabbe et al. (1999) published in Science caused a shock wave in the global scientific community because it indicated that even simple parameters such as motor activity and weight gain are site dependent (with the exception of gender differences) and thus not replicable. However, in the last 20 years, many new scientific discoveries have been made that contribute to a better understanding of the replication problems from one laboratory to another. Epigenetic events and microbiotic differences often contribute to significant trait and behavioral changes, even in genetically identical inbred strains (Blewitt and Whitelaw, 2013; Vuong et al., 2017; Chu et al., 2019). Furthermore, natural variabilities in stress resilience may also contribute to this problem (Kalinichenko et al., 2019). This is no different with humans. Monozygotic twins who are raised in different families and places can differ significantly in what they do and how they do it and how they cope with stressful situations. The environment shapes our behavior and stress coping strategies *via* epigenetic mechanisms, the immune state (Kalinichenko et al., 2019), and the environment-dependent settlement of microorganisms, especially in our intestines, that also have a considerable influence on our behavior and resilience or vulnerability to various diseases (Blewitt and Whitelaw, 2013; Vuong et al., 2017; Chu et al., 2019). These new findings provide an explanatory framework to better explain the variability that arises from measurements in different laboratories.

In summary, there are major statistical problems, poor methodology—the accuracy with which animal research is collected and described—and “method hopping,” as well as generalization challenges that contribute to the replication crisis in animal experiments. In addition to replication problems, several other phenomena contribute to translation failures from animal to human research.

## A Summary of Phenomena that Contribute to Translation Failures in Animal to Human Research

A publication bias for positive results contributes significantly to the lack of translation from animal to human research. This publication bias has been impressively demonstrated by Fanelli (2012). Fanelli compared original publications reporting positive results over a 20-year span and demonstrated a consistent positive publication bias in the field of neuroscience. Regardless of the research area and country of origin of the research, positive results were almost exclusively (>90% of all published studies) published in the preceding decades. Very similar pronounced publication biases for positive results were also found for other disciplines, such as pharmacology and psychiatry, and appears to be a global phenomenon in science (Fanelli, 2012).

Another source of translation failures is the incorrect choice of the model organism. Sydney Brenner—best known for his brilliant scientific quotes—concluded in his Nobel award speech “*Choosing the right organism for one’s research is as important as finding the right problems to work on*.” Convergent brain anatomy, complex behavioral repertoire, advanced cognitive capacities, and close genetic homology with humans make non-human primates by far the best model organism for behavioral neuroscience. However, the use of non-human primates for scientific purposes became an ethical issue in Western industrialized countries and today we are facing a situation where most of non-human primate research is done in China. Especially, advances in cloning of macaque monkeys allows now the generation of monkeys with uniform genetic backgrounds that are useful for the development of non-human primate models of human diseases (Liu et al., 2019).

For most studies in the field of behavioral neuroscience, a rodent model organism is the first choice, but a notable shift has occurred over the last two decades, with mice taking a more and more prominent role in biomedical science compared to rats (Ellenbroek and Youn, 2016; Yartsev, 2017). This shift is primarily driven by the availability of a huge number of transgenic mouse lines. However, for modeling complex behavior (e.g., social behavior) or pathophysiology (e.g., addictive behavior), the rat is the model organism that typically produces better translational information (Vengeliene et al., 2014; Ellenbroek and Youn, 2016; Spanagel, 2017). For example, whereas the majority of rats readily engage in social behavior, mice spend significantly less time interacting with a conspecific and many even find such interactions aversive. As a result, interactions between males are much rarer and, when they do occur, are more aggressive and territorial in nature. Furthermore, their social play-fighting involves only a small subset of what is exhibited by rats (Pellis and Pasztor, 1999). Clearly, social behavior in mice shows less face validity than that in rats. Nevertheless, a simple Medline search from 2002 to 2022 for the Keywords (social interaction) and (mice) has 2,400 hits compared to 1,700 hits for (social interaction) and (rat). In conclusion, despite the fact that rats are more social than mice and thus may better model human social behavior, researchers are preferring to use mice because more transgenic lines are available and housing costs are less.

In addition to the choice of the model organism, the employed animal test also has a great impact on translatability to the human situation. This is particularly evident when modeling aspects of complex pathological behavior as seen in different psychiatric conditions. The current DSM-5 and ICD-11 psychiatric diagnostic classification systems are based on clinical observations and patient symptom reports, and are inherently built on anthropomorphic terms. As diagnoses are made according to these classification systems worldwide, logic dictates that animal models of psychiatric disorders should also be based on DSM-5/ICD-11 criteria. Is this possible? Modeling the full spectrum of a mental disorder in humans is not possible in animals due to the high level of complexity. However, we can transfer anthropomorphic terminology to animal models with empirical, translatable and measurable parameters, and thus reliably examine at least some key criteria of a disease of interest in animal models—excellent examples for DSM-based animal models are provided for addictive behavior (Deroche-Gamonet and Piazza, 2014; Spanagel, 2017).

Further translation problems relate to pharmacotherapy development. Many preclinical studies aim to generate evidence that a new drug improves pathological behavior in a given animal model. However, the phenomenon of tolerance development—defined as a loss of efficacy with repeated drug exposure—is quite common but rarely assessed. Indeed a recent analysis indicates that many published preclinical efficacy studies in the field of behavioral neuroscience are conducted with acute drug administration only (Bespalov et al., 2016). Another limitation in the drug development process is that most preclinical drug testing in animals is conducted by intraperitoneal or subcutaneous administration while in humans the same treatment is planned to be oral. However, the pharmacokinetics of a given drug very much depends on the route of administration. If oral administration is already considered on the preclinical level appropriate dosing in humans can be better achieved, and it can be predicted early on if adequate therapeutic window exists.

Another problem that has yet to be discussed in the literature is the lack of a placebo effect in animal studies. Conditioned placebo effects, in particular placebo-induced analgesia, have been described in laboratory animals (Herrenstein, 1962; Keller et al., 2018). But when testing for pharmacological interventions in an animal model of a given psychiatric condition, a placebo effect cannot occur in laboratory animals unless they are first conditioned to the drug; however, this possibility is not given due to the experimental design). It can be assumed that the missing placebo effect in preclinical intervention studies leads to a considerable overestimation of the effect size in the translation in human clinical trials.

In summary, there are numerous phenomena that contribute to translation failures. It is not only the non-reproducibility of preclinical findings, but also a publication bias for positive results, the incorrect choice of the model organism, and the use of non-relevant disease models (for example not DSM-5/ICD11 conform) that result in translation failures in the clinical condition. With respect to preclinical psychopharmacotherapy development, tolerance phenomena are often not assessed, and the lack of identification of placebo effects in animal studies can lead to an overestimation of the effect size that can easily shrink due to large placebo effects common in neuropsychiatric trials (Scherrer et al., 2021).

In the second part of this perspective I will present a list of recommendations on how to counteract replication and translation failures.

## What Can Be Done to Improve Reproducibility in Animal Experimentation?

### Effect Size Estimation and Bayes Factors Are Alternatives to *P*-values

Is there an alternative to the inherent problem of a fickle *P*-value (Halsey et al., 2015) in exploratory animal studies that use a small sample size, typically in the range of *n* = 5–12? Not really, as exploratory animal research is performed under the limitation of lab resources and time, and must be also conducted within the 3R framework. Furthermore, the use of laboratory animals in biomedical research is a matter of intense public debate. Recent statistics indicate that about half of the western population would be in favor while the other half would oppose it (Petetta and Ciccocioppo, 2021). Therefore, the authorities that evaluate and approve the animal protocols are under pressure of this public and political debate and thus try to limit the number of animals to be employed.

What would help, however, is to report an effect size that gives quantitative information about the magnitude of the effect and its 95% confidence interval (CI), which indicates the uncertainty of that measure by presenting the range within which the true effect size is likely to lie (Michel et al., 2020). Indeed it has become more and more common practice to report effect sizes along with *P*-values. Reporting the effect size provides researchers with an estimate of how big the effect is, i.e., the difference between two groups. There are many ways to calculate the effect size—for a simple group comparison Cohen’s d is usually calculated. There is general agreement that *d* = 0.2 is a small effect, *d* = 0.5 a medium effect size, and *d* = 0.8 a large effect size. The calculation of the effect size with its 95% CI can help the researcher in deciding whether to continue with a study or abandon it because the findings are not reliable. If an exploratory experiment yields a highly significant effect (*P* < 0.01) with a small effect size (*d* = 0.2), one should be cautious to conduct further elaborate experiments. If an exploratory experiment yields a significant effect (*P* < 0.05) with a large effect size (*d* = 0.8), a better basis for further experiments is provided. Although these two scenarios of statistical analysis may help researchers to decide to continue or rather stop a study, the real advantage of describing effect sizes is that findings from several experiments can be combined with a meta-analysis to obtain more accurate effect size estimates.

Furthermore, one can augment or substitute a fickle *P*-value with the Bayes factor to inform on the relative levels of evidence for the null and alternative hypotheses; this approach is particularly appropriate for studies in which one wishes to continue collecting data until clear evidence for or against a working hypothesis has accrued (Halsey, 2019). The use of the Bayes factor is also starting to gain momentum and is easily calculable for a range of standard study designs (van de Schoot et al., 2021). Although in recent years Bayesian thinking and statistics are increasingly applied to many scientific fields, there is still a lack of knowledge and guidance on how to use this statistical approach, and most, scientific journals have yet to adapt their journal policies toward Bayesian statistics. However, even if we can augment or substitute a fickle *P*-value with a Bayes factor, it will not solve the inherent problem of using small sample sizes in exploratory research.

### Convergent Evidence From Cross-Species Studies Provide More Solid Ground for Replication

Alternatively to these different statistical approaches, a better assessment of reproducibility is obtained with convergent evidence, in which measurements obtained by different methods point to a similar finding. Convergent evidence requires more experiments with different methodological approaches that try to prove a working hypothesis. Although this clearly means more work, more experimental animals, and more time necessary to answer a research question, it provides more solid ground for replication. Convergent evidence can also be obtained by cross-species comparisons. Notably, convergent evidence that comes from cross-species neuroimaging and genetic studies provides solid evidence. Multi-modal neuroimaging for structural and functional measures is a standard methodology to study human psychopathology and the same measures can also be used in high-field scanners for experimental laboratory animals; if similar structural or functional brain signatures are obtained in humans and experimental animals, convergent cross-species evidence is provided. For example, converging evidence from diffusion tensor imaging measures in alcohol-dependent rats and humans show comparable persistent white matter alterations that evolve soon after the cessation of alcohol use (De Santis et al., 2019). This convergence now allows to study in alcohol-dependent rats the underlying molecular cause for the persistent white matter alterations, a methodological step that could not be done in humans. In terms of cross-species genetics, another example is a study using a genome-wide association meta-analysis (GWAS) of alcohol intake that demonstrates an association of a genetic variant in the ras-specific guanine-nucleotide releasing factor 2 (RASGRF2) gene, which encodes a protein that mediates activation of the ERK pathway. This association only occurred in males and it was further shown that male individuals showed a blunted mesolimbic dopamine response to alcohol-related stimuli (Stacey et al., 2012). Accordingly, alcohol intake was decreased in male but not female Rasgrf2 knockout mice and alcohol-induced dopamine release in the mesolimbic system was also blunted in male Rasgrf2 knockouts (Stacey et al., 2012). This cross-species genetic study shows convergent evidence for a sex-specific effect of a specific gene variant in alcohol intake and provides a plausible molecular mechanism.

### Preregistration Avoids P-Hacking and HARKing

**FIGURE 1:**
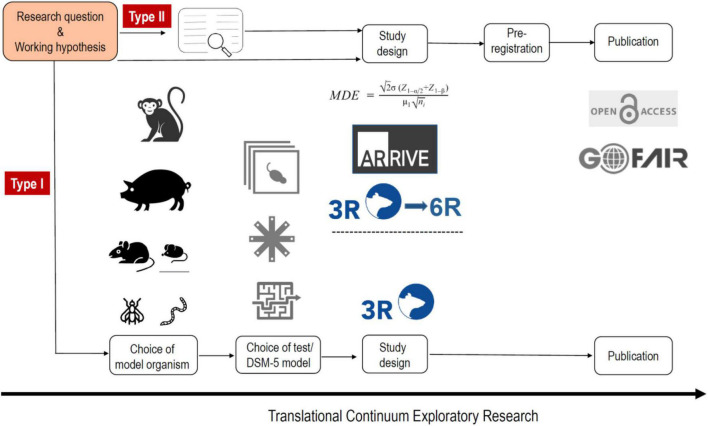
The workflow of an exploratory animal study type I and II. In the case of an exploratory study Type I, in which methodological new ground is broken the choice of model organism is of critical importance as well as the choice of behavioral test (which could be also a DSM-5-based behavioral model). The study design should be in accordance with the 3R principles, however, registration of the study design makes no sense, since experimental parameters are likely to be modified again and again in the course of the study until the new approach ultimately measures what is intended and can then be published. For a type II study the method is already established in a laboratory and is used to carry out new measurements. The study design can involve a power calculation (based on a systematic review), and is in accordance with the ARRIVE 2.0 guidelines and the 3R/6R principles. Pre-registration should be done and the publication irrespective if it reports negative or positive results should be open access and all digital data should be handled in accordance to the FAIR principles.

**FIGURE 2:**
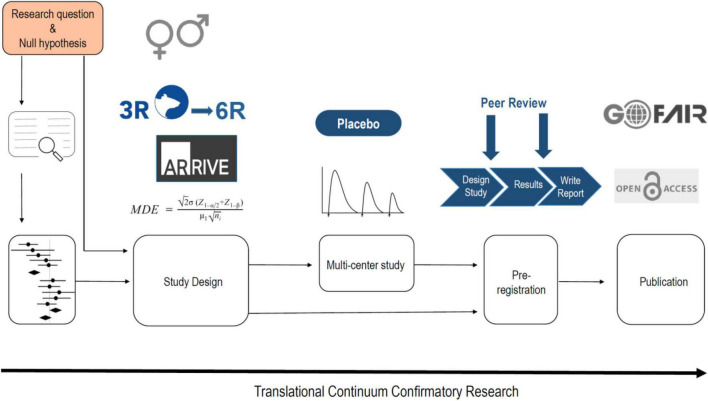
The workflow of a confirmatory animal study. If possible the null hypothesis should be based on a systematic review and even better for a quantitative statement on a meta-analysis. However, for most hypotheses it is not possible to perform a meta-analysis. The study design should include a power calculation, should adhere to the ARRIVE 2.0 guidelines and the 3R/6R principles, and should involve both sexes for generalization. For drug testing a multi-center study provides best translation and should consider tolerance development and a correction for the placebo effect. The study has to be pre-registered also in the form of a registered report. The publication should be open access and all digital data should be handled in accordance to the FAIR principles.

As indicated above, p-hacking and HARKing are common practices in science. A very simple way to avoid these phenomena is with pre-registration of the study design. Thus animal studies should be registered, much like the registration of clinical trials. There are already various platforms for this, e.g., www.animalstudyregistry.org or www.preclinicaltrials.eu (Pulverer, 2020). In the case of an exploratory study Type I, in which methodological new ground is broken, however, registration of the study design makes little sense, since experimental parameters are likely to be modified again and again in the course of the study until the new method ultimately measures what is intended. Registration only makes sense if a method is already established in a laboratory and is used to carry out new measurements (exploratory study) (Figure 1), or if previously published findings are to be confirmed (confirmatory study) (Kimmelman et al., 2014; Figure 2).

Confirmatory study designs can also published in the form of a Registered Report. This is a publication format that emphasizes the importance of the research question and the quality of the methodology through peer review prior to data collection. High-quality protocols are then tentatively accepted for publication if the authors use the registered methodology. The Center for Open Science in Charlottesville, United States already lists 295 scientific journals that offer the Registered Report Format.

### The ARRIVE Guidelines Lead to a Comprehensible Methodology

The ARRIVE Guidelines (Animal Research Reporting of *In Vivo* Experiments)^1^ are a well-established instrument for the transparent presentation of the methodology used. Precise reporting on study design, blinding, age and sex of the animals, and many more experimental factors are critical for future replication studies. Many journals have already implemented ARRIVE 2.0 in the guidelines for authors (Percie du Sert et al., 2020). These guidelines, which are attached to a submitted manuscript in the form of a checklist, lead to improved reporting on animal experiments and create transparency for both the editors of scientific journals and reviewers. For complex studies, especially in the case of confirmatory preclinical animal studies, professional help should also be sought and may be required in later FDA or EMA approval procedures. Thus, questions about the study design, statistical procedures and patent law implications should be clarified by professionals.^2^ The ARRIVE Guidelines and professional advice lead to a comprehensible methodology that enables other laboratories to use the method as well as replicate the resulting data. Unfortunately, ARRIVE guidelines are often adopted to a previously performed experiment. In order to improve this counterproductive conduct, ARRIVE 2.0 compliant study designs should be pre-registered.

Method hopping, the race for the newest technology, is of major concern when it comes to replication. It is a system-inherent problem of biomedical research in general and will only improve by changing the policies of high impact journals and evaluation procedures of high profile grant applications. However, each individual scientist is also responsible for his/her contribution to this system-inherent problem, as each scientist is free to decide to be a part of this race or to rely on a well-established technology. A skeptical view on method hopping should not hinder technological developments in the biomedical field, but a critical attitude toward the utility of new technologies results in a balance between the use of a well-established technology and new cutting-edge technology.

### Systematic Reviews and Meta-Analyses Lead to Generalized Conclusion

As previously stated, findings from animal studies, even when replicated, can ultimately only be generalized to a limited extent to the heterogeneous human situation. For example, gender-specific differences are common in psychiatric diseases. How can those biological differences be captured by animal experiments? The National Institutes of Health (NIH) released a policy in 2015 on sex as a biological variable, calling for researchers to factor sex into research designs, analyses, and reporting in animal studies. If NIH-funded researchers propose studying only one sex, they must provide “strong justification from the scientific literature, preliminary data, or other relevant considerations” to justify this (Clayton and Collins, 2014). This NIH driven policy applies more and more to global animal experimentation, and comparative studies on both sexes should be implemented in all exploratory type II studies and confirmatory studies. For exploratory studies type I, in which methodological new ground is broken, the focus on one sex is usually sufficient.

The generalization of variable and sometimes opposing results that arises from measurements in different laboratories can be obtained by systematic reviews and even quantified by meta-analyses. There is already a long tradition in clinical research of conducting systematic reviews and meta-analyses, which have become an evidence-based foundation of clinical research (Gurevitch et al., 2018). In 1976, the statistician Gene Glass coined the term “meta-analysis” to refer to “the statistical analysis of a large collection of analytical results from individual studies to integrate the results” (Glass, 1976; O’Rourke, 2007). In medicine, the meta-analysis has quickly established itself as an evidence-based instrument. Together with the more than 12,000 systematic reviews of the international research network Cochrane, essential building blocks for improved replication of clinical studies have been established. However, systematic reviews and meta-analysis in animal research have largely gone unnoticed by many researchers and have only recently gained momentum. An example on sex differences illustrates the power of doing a meta-analysis using animal data. It is assumed that behavioral repertoires of males and females may arise from differences in brain function. This may particularly apply to the neurochemistry of the mesolimbic dopamine system in reward processing. Thus, it is generally assumed that differences in basal dopamine levels in the nucleus accumbens and reward-induced dopamine-releasing properties are sex-specific (Müller, 2020). However, a recent meta-analysis that included 676 female and 1,523 male rats found no sex differences in basal levels of dopamine or dopaminergic response to rewards (Egenrieder et al., 2020). In conclusion, asking a research question and stating a working hypothesis accordingly should be based, if possible, on a systematic review or even meta-analysis. For confirmatory animal studies especially, but also to some extent exploratory studies type II, a systematic review or even meta-analysis should be considered (Figures 1, 2).

### Multi-Site Preclinical Confirmatory Trials as a New Module for Translating Animal Findings to a Heterogeneous Human Population

Furthermore, we can use the variability that arises from measurements in different laboratories for improved translation. Clinical trials are usually multi-center, and therefore the logical conclusion is that multi-center preclinical studies provide a better prediction of the clinical situation. Although the aforementioned study by Crabbe et al. (1999) provides an excellent example of a multi-site animal study, this methodology is still very rarely used. The reason for this rare use is that there is so far little guidance on how to conduct a preclinical confirmatory multi-site trial. As mentioned in Box 1, confirmatory studies are of particular interest for testing a drug’s clinical potential and restricting the advance of ineffective interventions into clinical testing (Kimmelman et al., 2014). This new preclinical module in the drug development process is currently being used for the first time as part of a European research program to study the effects of psychedelic drugs in an animal model of alcohol addiction.^3^ However, even if multi-site preclinical testing is very well-powered and probably the best approach for the translation of animal studies to humans, the challenge of heterogeneity seen in a human population remains. A preclinical study is usually performed in a specific outbred or inbred rodent strain. Although there is individual variability in behavioral measurement even in inbred strains, limited genetic and behavioral diversity in rodents fails to capture the phenotypic and genetic variability seen in human studies. One way to overcome this challenge is the use of the heterogeneous stock (HS) rats. These are highly recombinant animals, established by crossbreeding eight genetically diverse founder strains (Hansen and Spuhler, 1984; Solberg Woods and Palmer, 2019), resulting in a diversity that mimics the diversity found in the human population. Together with behavioral characterization, this allows for genetic analysis for any measurable behavioral or psychopathological quantitative trait by whole-genome sequencing of each individual. Combining hundreds of animals’ genetic data can thus feed a subsequent GWAS to identify gene variants contributing to a phenotype of interest. Moreover, using a new multidimensional data clustering strategy of large-scale data sets across different study sites can now be used to identify specific behavioral domains for disease vulnerability or resilience in rats (Allen et al., 2021). Therefore, it is recommended to use HS rats in confirmatory preclinical multi-site studies to mimic the human situation and provide additional genetic information under well-controlled environmental conditions.

**Box 1. Glossary**

**ARRIVE 2.0** The ARRIVE guidelines (Animal Research: Reporting of *In Vivo* Experiments) are a checklist of recommendations to improve the reporting of research involving animals—with the aim of increasing the quality and reliability of published research, and thereby increasing reproducibility of animal research (www.arriveguidelines.org). In addition see also the PREPARE guidelines (Planning Research and Experimental Procedures on Animals: Recommendations for Excellence) www.norecopa.no/prepare.

**Confirmatory animal study** Confirmatory animal studies resemble to a large degree clinical trials. In this type of study, an *a priori* hypothesis that is built on previously published results is stated, an adequate power calculation is made, a pre-specified experimental design is pre-registered, and all ARRIVE 2.0 and FAIR guidelines are strictly followed. Confirmatory studies aim less at elaborating theories or mechanisms of a drug’s action than rigorously testing a drug’s clinical potential and restricting the advance of ineffective interventions advanced into clinical testing (Kimmelman et al., 2014). In the best case scenario, confirmatory animal studies are performed in two or more laboratories, resembling a multi-site clinical trial design. Hence, **multi-site confirmatory preclinical studies** are a new module in the drug development chain and have a great potential to improve the translation of preclinical animal research to humans (Figure 2).

**Exploratory animal study Type I** In this type of study, scientifically and often methodologically novel ideas are developed, and experimental parameters must be modified again and again in the course of the study until the new method ultimately measures what is intended. Exploratory studies Type I are often driven by a loosely articulated working hypothesis or a hypothesis that evolves over the course of sequential experiments (see also HARKing). The sequence of individual experiments in exploratory studies and details of their design (including sample size, since effect sizes are unknown) are typically not established at the beginning of investigation. Exploratory animal studies Type I aim primarily at developing pathophysiological theories (Kimmelman et al., 2014; Figure 1).

**Exploratory study Typ**e **II** In this type of study, a method already established in a laboratory to carry out new measurements is used to break new scientific ground. In this case, a working hypothesis is clearly stated, a power calculation is made because effect sizes are usually known from previous experiments, pre-registration is recommended, and ARRIVE 2.0 and FAIR guidelines should be followed (Figure 1).

**FAIR** In 2016, the FAIR Guiding Principles for scientific data were published (Wilkinson et al., 2016). FAIR improves the **F**indability, **A**ccessibility, **I**nteroperability, and **R**euse of digital datasets (www.go-fair.org). Initially FAIR focused on datasets from human research and trials; very recently the guidelines were also adapted to preclinical research (Briggs et al., 2021).

**HARCKing** Hypothesizing After the Results are Known.

**Method hopping** Method hopping refers to the race for the newest technology and has the potential to produce unreliable measurements.

**P-hacking** The selective reporting of significant data. Data analysis is performed in such a way as to find patterns in data that can be presented as statistically significant, thus dramatically increasing the risk of false positives.

**3R** Russell and Burch introduced the 3R principles more than 60 years ago (Tannenbaum and Bennett, 2015). The 3Rs (Replacement, Reduction, and Refinement) have become the guiding principles for the ethical use of animals in research (www.nc3rs.org.uk).

**6R** Although the 3Rs provide the ethical principle of animal research, animal welfare alone does not suffice to make animal research ethical if the research does not have sufficient scientific value. Therefore, Strech and Dirnagl (2019) introduced Robustness, Registration and Reporting, in addition to the 3Rs, all of which aim to safeguard and increase the scientific value and reproducibility of animal research.

Multi-site preclinical confirmatory trials aim for rigorously testing a drug’s clinical potential and restricting the advance of ineffective interventions advanced into clinical testing (Kimmelman et al., 2014). As said the phenomenon of tolerance development is quite common but rarely assessed (Bespalov et al., 2016). Therefore, it is essential to plan within the study design chronic intermittent treatment schedules, similar to the application in clinical trials, to study the occurrence of tolerance. In addition to studying tolerance, the pronounced placebo effect usually seen in a clinical psychiatric trial has to be also considered in multi-site preclinical confirmatory trials. Since a placebo effect does not occur in those animal studies one has to correct for the placebo effect otherwise an overestimation of the effect size is achieved. Especially, in less severe study populations a medium effect size for placebo is often reported (Scherrer et al., 2021). Therefore, if the effect size in a multi-site preclinical confirmatory trials for drug testing is not at least large (*d* ≥ 0.8) it is unlikely that a later preclinical trial that builds on those preclinical findings will yield significant results.

Multi-site preclinical confirmatory trials are cost-intensive and require a well harmonized collaborative effort. Moreover, there are only very limited funding schemes that would support such an approach. An alternative is to study a large animal cohort by splitting an experiment into several “mini-experiments” spread over different time points a few weeks apart. Indeed, such a “mini-experiment” design in comparison to a conventionally standardized design, according to which all animals are tested at one specific point in time improved the reproducibility and accurate detection of exemplary treatment effects (von Kortzfleisch et al., 2020).

### Publishing Negative Results to Counteract the Publication Bias for Positive Results

A large proportion of animal experimentation results in negative results that do not supporting the working hypothesis. These negative results are usually not published, resulting in a publication bias. However, publishing negative results is easier said than done, as it is difficult to convince reviewers of the validity of negative results. Reviewers often require more control experiments and converging evidence to support the validity of negative findings in the peer-review process than in the review of positive findings. Moreover, scientific journals have little interest in publishing negative findings (Bespalov et al., 2019). In return, Springer Publisher founded the Journal of Negative Results in BioMedicine back in 2002. The journal was not able to assert itself in the “market,” did not receive an impact factor, and was discontinued in 2017. This problem has been recognized, as major journals such as Nature trumpet “Highlight negative results to improve science” (Mehta, 2019), but in fact there is little movement in the publication landscape. Instead of HARKing and p-hacking and thus turning a negative result into a false positive, negative results should be published as they are.

### Open Science for Scientific Data and Publications

There is now a strong open science movement to publish results, either positive or negative, as open access, and scientific data should be handled by the FAIR Guiding Principles that act within the open science framework. Although the open science movement has great advocacy by many stakeholders, some criticism is also of note. Every transformation process—such as the open science movement—is complex and requires time and financial resources. The pendulum often swings too far during a transformation process and good intentions are quickly misguided. This process is best described by the implementation of Open Access publications. Free access to scientific literature and the consequent accessibility of primary and metadata is certainly a noble goal. As a result, everyone has access to scientific literature, and the journal crisis—the annual increase in prices for scientific journals with stagnant or even declining budgets—can be eliminated. There is a cost to that goal, but who foots the bill? In Germany, for example, an alliance of various scientific organizations initiated the DEAL project a few years ago. New contract models were negotiated nationwide with the three major scientific publishers: Elsevier, Springer Nature, and Wiley. DEAL was completed in 2020 and one would think that open access publishing in Germany would be a foregone conclusion. Not even close. At almost all universities, it is largely unclear who is required to bear the costs, which are increasingly being passed on to the individual working groups. Incidentally, the prices are rising at the same rate, and the publishers are earning even more than before. In the beginning of 2022, Nature Neuroscience released their Article Processing Charge (APC) to be paid for gold access, which is US$11,390. Publishing scientific findings is and will remain big business, and whether the Open Access movement will counteract this or even promote economization can only be assessed in the next 10 years. Basically, scientific publishing is a perverted world anyway: imagine that an author painstakingly writes a novel, several editors proofread it free of charge and the publisher then charges the author a high fee for publication, so that the book is now freely accessible. Unthinkable in the free economy, everyday business in science.

## Summary of Points for Recommendation to Improve Reproducibility and Translation

In Box 2 ten points of recommendation are summarized that will help to improve reproducibility and translation of animal experimentation. Most of these recommendations are also part of the new 6R framework that enlarges the scope of the widely established 3R framework for the ethical use of animals in research by three additional guiding principles that are Robustness, Registration and Reporting, all of which aim to safeguard and increase the scientific value of animal research (Strech and Dirnagl, 2019).

**Box 2. [b] Ten points of recommendation to improve reproducibility and translation**

(i) Prior to planning an actual study, a systematic review or potential preclinical meta-analysis should be considered.
(ii) An *a priori* power calculation should be carried out.
(iii) The experimental study protocol should be pre-registered.
(iv) The execution of the study should be in accordance with the most recent ARRIVE guidelines. In addition to conventional statistics Bayes values and effect size estimation should be considered.
(v) When planning the study, the generalizability of the data to be collected should also be considered (e.g., sex or age differences).
(vi) “Method-hopping” should be avoided, meaning that it is not necessary to use the most advanced technology but rather to have the applied methodology under control.
(vii) National or international networks should be considered to obtain convergent evidence or to carry out a multicenter preclinical confirmatory study. The latter aimed at conducting drug testing should also consider tolerance development and correction for placebo effects.
(viii) Animal models that capture DSM-5 or ICD-11 criteria should be considered in the context of research on psychiatric disorders.
(ix) Raw data of publication should be made publicly available and digital datasets should be in accordance with the FAIR Guiding Principles for scientific data management.
(x) Negative findings should be published to counteract publication bias.

## Conclusion

Science has long been regarded as “self-correcting,” given that it is founded on the replication of earlier work. However, in recent decades the checks and balances that once ensured scientific fidelity have been challenged in the entire biomedical field. This has compromised the ability of today’s researchers to reproduce others’ findings (Collins and Tabak, 2014), and has also resulted in many translation failures. This perspective article outlines a list of reasons responsible, to a large extent, for a lack of reproducibility in animal studies and difficulties in translation from animals to humans. This perspective article also provides recommendations to improve reproducibility and translation. However, even if the 10 points of recommendation listed in Box 2 are implemented, a translation error can always occur. The decisive question shall then be: Why did a translation error occur? Here a prominent example of a translation error—the development of corticotropin-releasing factor (CRF) receptor 1 antagonists for alcohol addiction and other stress-related psychiatric conditions—is provided.

Convincing evidence from animal studies demonstrated an up-regulated CRF and CRF-R1 expression within the amygdala that underlie the increased behavioral sensitivity to stress following development of alcohol dependence (Heilig and Koob, 2007). Animal studies also suggested that CRF activity plays a major role in mediating relapse provoked by stressors, as well as escalation of drinking in alcohol dependent rats (Heilig and Koob, 2007). These findings led to the expectation that brain penetrant CRF-R1 antagonists would block stress-induced craving and relapse in people with alcohol addiction. Big-Pharma invested hundreds of millions in drug development programs for CRF-R1 antagonists and subsequent clinical trials. However, clinical trials in alcohol dependent patients have not supported the expectations (Kwako et al., 2015; Schwandt et al., 2016). Other clinical trials with CRF-R1 antagonists for stress-related psychiatric conditions also failed. This includes negative trials for depression (Binneman et al., 2008), anxiety (Coric et al., 2010), and post-traumatic stress disorder (Dunlop et al., 2017). This translation failure, however, was the results of a publication bias for positive results and the ignorance of earlier animal studies that indicated that CRF-R1 antagonists will not work in clinical trials. Already in 2002 it was shown that CRF-R1 knockout mice show an escalation in alcohol drinking (Sillaber et al., 2002) and that CRF-R1 antagonists do not reduce drinking in a relapse model (Molander et al., 2012). Moreover, CRF-R1 can produce bidirectional effects on motivational behavior, depending on the neuronal population in which this receptor is expressed (Refojo et al., 2011). These studies with negative or even opposing results explain the observed null effects of antagonists on stress-induced disorders and one wonders why those results were not considered at the time when cost-intensive clinical trials were initiated.

One can learn from translation errors. For this purpose, however, a critical dialogue between basic researcher, pre-clinicians and clinicians is of essential importance. There is only a crisis if we cannot find an explanation for the lack of replication and/or a translation failure.

## Data Availability Statement

The original contributions presented in the study are included in the article, further inquiries can be directed to the corresponding author/s.

## Author Contributions

The author confirms being the sole contributor of this work and has approved it for publication.

## Conflict of Interest

The author declares that the research was conducted in the absence of any commercial or financial relationships that could be construed as a potential conflict of interest.

## Publisher’s Note

All claims expressed in this article are solely those of the authors and do not necessarily represent those of their affiliated organizations, or those of the publisher, the editors and the reviewers. Any product that may be evaluated in this article, or claim that may be made by its manufacturer, is not guaranteed or endorsed by the publisher.

## Footnotes

1
www.arriveguidelines.org

2
www.paasp.net

3
www.psialc.org

## References

[B1] Allen C. Kuhn B. N. Cannella N. Crow A. D. Roberts A. T. Lunerti V. (2021). Network-Based discovery of opioid use vulnerability in rats using the bayesian stochastic block model. *Front. Psychiatry* 12:745468. 10.3389/fpsyt.2021.745468 34975564 PMC8718996

[B2] Baker M. (2016). 1,500 scientists lift the lid on reproducibility. *Nature* 533 452–454. 10.1038/533452a 27225100

[B3] Blewitt M. Whitelaw E. (2013). The use of mouse models to study epigenetics. *Cold Spring Harb. Perspect. Biol.* 5:a017939. 10.1101/cshperspect.a017939 24186070 PMC3809579

[B4] Bespalov A. Steckler T. Altevogt B. Koustova E. Skolnick P. Deaver D. (2016). Failed trials for central nervous system disorders do not necessarily invalidate preclinical models and drug targets. *Nat. Rev. Drug Discov.* 15:516. 10.1038/nrd.2016.88 27312728

[B5] Bespalov A. Steckler T. Skolnick P. (2019). Be positive about negatives-recommendations for the publication of negative (or null) results. *Eur. Neuropsychopharmacol.* 29 1312–1320. 10.1016/j.euroneuro.2019.10.007 31753777

[B6] Binneman B. Feltner D. Kolluri S. Shi Y. Qiu R. Stiger T. (2008). A 6-week randomized, placebo-controlled trial of CP-316,311 (a selective CRH1 antagonist) in the treatment of major depression. *Am. J. Psychiatry* 165 617–620. 10.1176/appi.ajp.2008.07071199 18413705

[B7] Briggs K. Bosc N. Camara T. Diaz C. Drew P. Drewe W. C. (2021). Guidelines for FAIR sharing of preclinical safety and off-target pharmacology data. *ALTEX* 38 187–197. 10.14573/altex.2011181 33637997

[B8] Button K. S. Ioannidis J. P. Mokrysz C. Nosek B. A. Flint J. Robinson E. S. (2013). Power failure: why small sample size undermines the reliability of neuroscience. *Nat. Rev. Neurosci.* 14 365–376. 10.1038/nrn3475 23571845

[B9] Chu C. Murdock M. H. Jing D. Won T. H. Chung H. Kressel A. M. (2019). The microbiota regulate neuronal function and fear extinction learning. *Nature* 574 543–548. 10.1038/s41586-019-1644-y 31645720 PMC6818753

[B10] Clayton J. A. Collins F. S. (2014). Policy: NIH to balance sex in cell and animal studies. *Nature* 509 282–283. 10.1038/509282a 24834516 PMC5101948

[B11] Collins F. S. Tabak L. A. (2014). Policy: NIH plans to enhance reproducibility. *Nature* 505 612–613. 10.1038/505612a 24482835 PMC4058759

[B12] Coric V. Feldman H. H. Oren D. A. Shekhar A. Pultz J. Dockens R. C. (2010). Multicenter, randomized, double-blind, active comparator and placebo-controlled trial of a corticotropin-releasing factor receptor-1 antagonist in generalized anxiety disorder. *Depress. Anxiety* 27 417–425. 10.1002/da.20695 20455246

[B13] Crabbe J. C. Wahlsten D. Dudek B. C. (1999). Genetics of mouse behavior: interactions with laboratory environment. *Science* 284 1670–1672. 10.1126/science.284.5420.1670 10356397

[B14] De Santis S. Bach P. Pérez-Cervera L. Cosa-Linan A. Weil G. Vollstädt-Klein S. (2019). Microstructural white matter alterations in men with alcohol use disorder and rats with excessive alcohol consumption during early abstinence. *JAMA Psychiatry* 76 749–758. 10.1001/jamapsychiatry.2019.0318 30942831 PMC6583663

[B15] Deroche-Gamonet V. Piazza P. V. (2014). Psychobiology of cocaine addiction: contribution of a multi-symptomatic animal model of loss of control. *Neuropharmacology* 76(Pt B) 437–449. 10.1016/j.neuropharm.2013.07.014 23916478

[B16] Dunlop B. W. Binder E. B. Iosifescu D. Mathew S. J. Neylan T. C. Pape J. C. (2017). Corticotropin-releasing factor receptor 1 antagonism is ineffective for women with posttraumatic stress disorder. *Biol. Psychiatry* 82 866–874. 10.1016/j.biopsych.2017.06.024 28793974 PMC5683912

[B17] Egenrieder L. Mitricheva E. Spanagel R. Noori H. R. (2020). No basal or drug-induced sex differences in striatal dopaminergic levels: a cluster and meta-analysis of rat microdialysis studies. *J. Neurochem.* 152 482–492. 10.1111/jnc.14911 31705667

[B18] Ellenbroek B. Youn J. (2016). Rodent models in neuroscience research: is it a rat race? *Dis. Model Mech.* 9 1079–1087. 10.1242/dmm.026120 27736744 PMC5087838

[B19] Fanelli D. (2009). How many scientists fabricate and falsify research? A systematic review and meta-analysis of survey data. *PLoS One* 4:e5738. 10.1371/journal.pone.0005738 19478950 PMC2685008

[B20] Fanelli D. (2012). Negative results are disappearing from most disciplines and countries. *Scientometrics* 90 891–904.

[B21] Glass G. V. (1976). Primary, secondary and meta-analysis of research. *Educ. Res.* 10 3–8.

[B22] Gurevitch J. Koricheva J. Nakagawa S. Stewart G. (2018). Meta-analysis and the science of research synthesis. *Nature* 555 175–182. 10.1038/nature25753 29517004

[B23] Halsey L. G. (2019). The reign of the p-value is over: what alternative analyses could we employ to fill the power vacuum? *Biol. Lett.* 15:20190174. 10.1098/rsbl.2019.0174 31113309 PMC6548726

[B24] Halsey L. G. Curran-Everett D. Vowler S. L. Drummond G. B. (2015). The fickle P value generates irreproducible results. *Nat. Methods* 12 179–185. 10.1038/nmeth.3288 25719825

[B25] Hansen C. Spuhler K. (1984). Development of the National Institutes of Health genetically heterogeneous rat stock. *Alcohol. Clin. Exp. Res.* 8 477–479. 10.1111/j.1530-0277.1984.tb05706.x 6391259

[B26] Head M. L. Holman L. Lanfear R. Kahn A. T. Jennions M. D. (2015). The extent and consequences of p-hacking in science. *PLoS Biol.* 13:e1002106. 10.1371/journal.pbio.1002106 25768323 PMC4359000

[B27] Heilig M. Koob G. F. (2007). A key role for corticotropin-releasing factor in alcohol dependence. *Trends Neurosci.* 30 399–406. 10.1016/j.tins.2007.06.006 17629579 PMC2747092

[B28] Heinz A. Kiefer F. Smolka M. N. Endrass T. Beste C. Beck A. (2020). Addiction research consortium: losing and regaining control over drug intake (ReCoDe)-From trajectories to mechanisms and interventions. *Addict. Biol.* 25:e12866. 10.1111/adb.12866 31859437

[B29] Herrenstein R. J. (1962). Placebo effect in the rat. *Science* 138 677–678. 10.1126/science.138.3541.677 13954106

[B30] Kalinichenko L. S. Kornhuber J. Müller C. P. (2019). Individual differences in inflammatory and oxidative mechanisms of stress-related mood disorders. *Front. Neuroendocrinol.* 55:100783. 10.1016/j.yfrne.2019.100783 31415777

[B31] Keller A. Akintola T. Colloca L. (2018). Placebo analgesia in rodents: current and future research. *Internat. Rev. Neurobiol.* 138 1–15. 10.1016/bs.irn.2018.02.001 PMC5918296 29681320

[B32] Kerr N. L. (1998). HARKing: hypothesizing after the results are known. *Pers. Soc. Psychol. Rev.* 2 196–217. 10.1207/s15327957pspr0203_4 15647155

[B33] Kilkenny C. Parsons N. Kadyszewski E. Festing M. F. Cuthill I. C. Fry D. (2009). Survey of the quality of experimental design, statistical analysis and reporting of research using animals. *PLoS One* 4:e7824. 10.1371/journal.pone.0007824 19956596 PMC2779358

[B34] Kimmelman J. Mogil J. S. Dirnagl U. (2014). Distinguishing between exploratory and confirmatory preclinical research will improve translation. *PLoS Biol.* 12:e1001863. 10.1371/journal.pbio.1001863 24844265 PMC4028181

[B35] Kwako L. E. Spagnolo P. A. Schwandt M. L. Thorsell A. George D. T. Momenan R. (2015). The corticotropin releasing hormone-1 (CRH1) receptor antagonist pexacerfont in alcohol dependence: a randomized controlled experimental medicine study. *Neuropsychopharmacology* 40 1053–1063. 10.1038/npp.2014.306 25409596 PMC4367465

[B36] Liu Z. Cai Y. Liao Z. Xu Y. Wang Y. Wang Z. (2019). Cloning of a gene-edited macaque monkey by somatic cell nuclear transfer. *Natl. Sci. Rev.* 6 101–108. 10.1093/nsr/nwz003 34691835 PMC8291622

[B37] Mehta D. (2019). Highlight negative results to improve science. *Nature* 10.1038/d41586-019-02960-3 [Online ahead of print] 33009522

[B38] Michel M. C. Murphy T. J. Motulsky H. J. (2020). New author guidelines for displaying data and reporting data analysis and statistical methods in experimental biology. *J. Pharmacol. Exp. Ther.* 372 136–147. 10.1124/jpet.119.264143 31884418

[B39] Molander A. Vengeliene V. Heilig M. Wurst W. Deussing J. M. Spanagel R. (2012). Brain-specific inactivation of the Crhr1 gene inhibits post-dependent and stress-induced alcohol intake, but does not affect relapse-like drinking. *Neuropsychopharmacology* 37 1047–1056. 10.1038/npp.2011.297 22113086 PMC3280644

[B40] Müller C. P. (2020). Everything you always wanted to know about sex and dopamine, but were afraid to ask: an Editorial for ‘No basal or drug-induced sex differences in striatal dopaminergic levels: a cluster and metaanalysis of rat microdialysis studies’ on page 482. *J. Neurochem*. 152 422–424. 10.1111/jnc.14916 31792978

[B41] Open Science Collaboration. (2015). Estimating the reproducibility of psychological science. *Science* 349:aac4716. 10.1126/science.aac4716 26315443

[B42] O’Rourke K. (2007). An historical perspective on meta-analysis: dealing quantitatively with varying study results. *J. R. Soc. Med.* 100 579–582. 10.1177/0141076807100012020 18065712 PMC2121629

[B43] Pellis S. M. Pasztor T. J. (1999). The developmental onset of a rudimentary form of play fighting in C57 mice. *Dev. Psychobiol.* 34 175–182. 10204093

[B44] Percie du Sert N. Hurst V. Ahluwalia A. Alam S. Avey M. T. Baker M. (2020). The ARRIVE guidelines 2.0: updated guidelines for reporting animal research. *PLoS Biol.* 18:e3000410. 10.1371/journal.pbio.3000410 32663219 PMC7360023

[B45] Perrin S. (2014). Preclinical research: make mouse studies work. *Nature* 507 423–425. 10.1038/507423a 24678540

[B46] Petetta F. Ciccocioppo R. (2021). Public perception of laboratory animal testing: historical, philosophical, and ethical view. *Addict. Biol.* 26:e12991. 10.1111/adb.12991 33331099 PMC9252265

[B47] Popper K. (1935). *Logik der Forschung Zur Erkenntnistheorie der modernen Naturwissenschaft*. Vienna: Springer, 54, 1–3. 10.1007/s10838-020-09531-5

[B48] Pulverer B. (2020). Registered animal studies and null data. *EMBO Rep.* 21:e49868. 10.15252/embr.201949868 31867857 PMC6944902

[B49] Refojo D. Schweizer M. Kuehne C. Ehrenberg S. Thoeringer C. Vogl A. M. (2011). Glutamatergic and dopaminergic neurons mediate anxiogenic and anxiolytic effects of CRHR1. *Science* 333 1903–1907. 10.1126/science.1202107 21885734

[B50] Scherrer B. Guiraud J. Addolorato G. Aubin H. J. de Bejczy A. Benyamina A. (2021). Baseline severity and the prediction of placebo response in clinical trials for alcohol dependence: a meta-regression analysis to develop an enrichment strategy. *Alcohol. Clin. Exp. Res.* 45 1722–1734. 10.1111/acer.14670 34418121 PMC9291112

[B51] Schwandt M. L. Cortes C. R. Kwako L. E. George D. T. Momenan R. Sinha R. (2016). The CRF1 antagonist verucerfont in anxious alcohol-dependent women: translation of neuroendocrine, but not of anti-craving effects. *Neuropsychopharmacology* 41 2818–2829. 10.1038/npp.2016.61 27109623 PMC5061889

[B52] Serra-Garcia M. Gneezy U. (2021). Nonreplicable publications are cited more than replicable ones. *Sci. Adv.* 7:eabd1705. 10.1126/sciadv.abd1705 34020944 PMC8139580

[B53] Sillaber I. Rammes G. Zimmermann S. Mahal B. Zieglgänsberger W. Wurst W. (2002). Enhanced and delayed stress-induced alcohol drinking in mice lacking functional CRH1 receptors. *Science* 296 931–933. 10.1126/science.1069836 11988580

[B54] Solberg Woods L. C. Palmer A. A. (2019). Using heterogeneous stocks for fine-mapping genetically complex traits. *Methods Mol. Biol.* 2018 233–247. 10.1007/978-1-4939-9581-3_11 31228160 PMC9121584

[B55] Spanagel R. (2017). Animal models of addiction. *Dialogues Clin. Neurosci.* 19 247–258. 10.31887/DCNS.2017.19.3 29302222 PMC5741108

[B56] Stacey D. Bilbao A. Maroteaux M. Jia T. Easton A. C. Longueville S. (2012). RASGRF2 regulates alcohol-induced reinforcement by influencing mesolimbic dopamine neuron activity and dopamine release. *Proc. Natl. Acad. Sci. U.S.A.* 109 21128–21133. 10.1073/pnas.1211844110 23223532 PMC3529066

[B57] Strech D. Dirnagl U. (2019). 3Rs missing: animal research without scientific value is unethical. *BMJ Open Sci.* 3:e000035. 10.1136/bmjos-2018-000048 35047678 PMC8647585

[B58] Tannenbaum J. Bennett T. (2015). Russell and Burch’s 3Rs then and now: the need for clarity in definition and purpose. *J. Am. Assoc. Lab Anim. Sci.* 54 120–132. 25836957 PMC4382615

[B59] van de Schoot R. Depaoli S. King R. Kramer B. Märtens K. Mahlet G. (2021). Bayesian statistics and modelling. *Nat. Rev. Methods Primers* 1:16. 10.1038/s43586-020-00001-2

[B60] Vengeliene V. Bilbao A. Spanagel R. (2014). The alcohol deprivation effect model for studying relapse behavior: a comparison between rats and mice. *Alcohol* 48 313–320. 10.1016/j.alcohol.2014.03.002 24811155

[B61] von Kortzfleisch V. T. Karp N. A. Palme R. Kaiser S. Sachser N. Richter S. H. (2020). Improving reproducibility in animal research by splitting the study population into several ‘mini-experiments’. *Sci. Rep.* 10:16579. 10.1038/s41598-020-73503-4 33024165 PMC7538440

[B62] Vuong H. E. Yano J. M. Fung T. C. Hsiao E. Y. (2017). The microbiome and host behavior. *Annu. Rev. Neurosci.* 40 21–49. 10.1146/annurev-neuro-072116-031347 28301775 PMC6661159

[B63] Wilkinson M. D. Dumontier M. Aalbersberg I. J. Appleton G. Axton M. Baak A. (2016). The FAIR Guiding Principles for scientific data management and stewardship. *Sci. Data* 3:160018. 10.1038/sdata.2016.18 26978244 PMC4792175

[B64] Yartsev M. M. (2017). The emperor’s new wardrobe: rebalancing diversity of animal models in neuroscience research. *Science* 358 466–469. 10.1126/science.aan8865 29074765

